# Microstructure Evolution and Localized Corrosion Susceptibility of an Al-Zn-Mg-Cu-Zr 7xxx Alloy with Minor Cr Addition

**DOI:** 10.3390/ma16030946

**Published:** 2023-01-19

**Authors:** Chijioke Kenneth Akuata, Feliksianus Robby Gunawan, Piyada Suwanpinij, Daniela Zander

**Affiliations:** 1 Chair of Corrosion and Corrosion Protection, Foundry Institute, Division of Materials Science and Engineering, RWTH Aachen University, Intzestraße 5, 52072 Aachen, Germany; 2 The Sirindhorn International Thai-German Graduate School of Engineering (TGGS), King Mongkut’s University of Technology North Bangkok (KMUTNB), 1518 Pracharat 1 Road, Wongsawang, Bangsue, Bangkok 10800, Thailand; 3 Leibniz-Institute für Werkstofforientierte Technologien—IWT, Badgasteiner Straße, 3, 28359 Bremen, Germany; 4 Deutsches Zentrum für Luft- und Raumfahrt, German Aerospace Center, Institute of Materials Physics in Space, Linder Höhe, 51147 Cologne, Germany

**Keywords:** Al-Zn-Mg-Cu-Zr alloys, microstructure evolution, Cr addition, recrystallization, electron backscatter diffraction (EBSD), scanning transmission electron microscopy (STEM), localized corrosion

## Abstract

Microstructure optimization of Al-Zn-Mg-Cu-Zr aluminum alloys, particularly through recrystallization inhibition, for improved strength and corrosion resistance properties has been an important consideration in alloy development for aerospace applications. Addition of rare earth elements, sometimes combined with Cr, has been found to be beneficial in this regard. In this study, the role of a single addition of 0.1 wt.% Cr on microstructure evolution of an Al-Zn-Mg-Cu-Zr (7449) alloy during processing was systematically investigated by optical light microscopy, scanning electron microscopy, electron backscatter diffraction and scanning transmission electron microscopy. Susceptibility to localized corrosion after aging to T4, T6 and T76 conditions was evaluated by potentiodynamic polarization (PDP) measurements and an intergranular corrosion (IGC) test. A decrease in recrystallized fraction with 0.1 wt.% Cr addition was observed, which is attributed to the formation of Cu- and Zn-containing E (Al_18_Mg_3_Cr_2_) dispersoids and the larger as-cast grain size. Moreover, the depletion of alloying elements from solid solution due to the formation of the Cu- and Zn-containing E (Al_18_Mg_3_Cr_2_) dispersoids and *η* Mg(Zn,Cu,Al)_2_ phase at its interface affects grain-boundary precipitation. The observed decrease in localized corrosion susceptibility with minor Cr addition is correlated with the microstructure and equally discussed.

## 1. Introduction

Al-Zn-Mg-Cu (7xxx) aluminum alloys find their application in lightweight design in the aerospace industry due to their superior specific strength, moderate corrosion resistance properties and excellent mechanical properties [1,2,3]. They are, however, characterized by a complex microstructure often developed at the various processing steps, viz. solidification, homogenization, hot forming, solution heat treatment and artificial aging, which affects the final mechanical and corrosion properties. One major factor with significant influence on the microstructure is the alloy chemistry. Alloying elements, whether intentionally added to achieve desired properties or inevitably present as impurities, could result in the formation of intermetallic particles during ingot solidification and post-casting processing.

Post-casting processing of 7xxx alloys proceeds with the homogenization heat treatment to dissolve coarse interdendritic eutectic phases formed during solidification, eliminate microsegregation and form fine dispersoids that inhibit recrystallization during subsequent ingot processing [4,5]. Undissolved coarse constituents, particularly the Fe- and Si-rich phases which are insoluble at the homogenization temperature, exert deleterious effects on alloy properties. Dynamic recrystallization through particle stimulated nucleation (PSN) mechanism, which results in new strain-free grain formation [6,7,8], increased localized corrosion susceptibility [9,10], lower fracture toughness and ductility [11,12], have been associated with the presence of intermetallic particles. 

Control of recrystallization and grain growth, which is important for improving mechanical and corrosion resistance properties, is done by trace addition of elements such as Mn, Cr and Zr [13,14], which form fine dispersoids. Among these, Zr is a preferred choice due to its ability to form fine coherent Al_3_Zr dispersoids that are less quench-sensitive. However, combined addition of Zr with rare earth elements such as Sc and Er, to mention a select few, can form fine coherent dispersoids with high thermal stability and enhanced recrystallization inhibition properties [15]. Cr addition, on the other hand, results in the formation of E (Al_18_Mg_3_Cr_2_) dispersoids, which are large and incoherent with the Al-matrix, and therefore exhibit lower recrystallization inhibition properties compared to Al_3_Zr, and results in a relatively high quench sensitivity effect [16,17]. Nonetheless, Cr is good to use for grain structure control in commercial 7xxx alloys, such as 7075 and 7475 [18]. 

Recent studies on the combined addition of Cr and a rare earth element in an Al-Zn-Mg-Cu-Zr alloy have shown improved recrystallization inhibition, which resulted in enhanced mechanical and corrosion resistance properties [19,20,21]. Fang et al. [22] attributed this to the formation of coherent Cr-containing Al_3_(Zr, Er) dispersoids, formed with combined addition of Cr and Er. In a different investigation, Liao et al. [23] reported that the addition of 0.1 wt.% Cr in an Al-8.54Zn-2.41Mg-1.3Cu-0.16Zr-0.3Yb alloy results in the formation of (Al,Cr)_3_(Zr,Yb) dispersoids, which improved recrystallization inhibition and localized corrosion resistance properties. Furthermore, addition of Cr in an Al-Zr binary system results in the conversion of DO23 Al_3_Zr to L12 Al_3_Zr with improved thermal stability [24,25,26,27]. Despite these findings, there is limited information on the effect of minor, single Cr addition with no rare earth element addition on the recrystallization and localized corrosion properties of a Zr-containing commercial 7xxx alloy. 

Therefore, this work aims to systematically understand the role of minor Cr addition, with no rare earth element addition, on the microstructure evolution of an Al-Zn-Mg-Cu-Zr alloy during processing and the resulting localized corrosion susceptibility in different aging conditions.

## 2. Materials and Methods

### 2.1. Thermodynamic Simulation

Thermophysical modeling of the Al-Zn-Mg-Cu-Zr-Cr multicomponent alloy system was performed to determine the optimum Cr composition for the formation of dispersoids without an additional primary phase formation. Thermodynamic equilibrium simulation was carried out using the commercial Thermo-Calc^®^ software with the TTAL6 aluminum database. Phase diagram and phase equilibrium simulations were performed with the composition of 7xxx alloy, as outlined in Section 2.2.

### 2.2. Alloy Processing

The materials used in this investigation are Al-Zn-Mg-Cu-Zr aluminum alloys within the composition range of 7449 aluminum alloy. To investigate the influence of Cr, 0.1 wt.% Cr was added using AlCr20 master alloy. This amount was chosen based on the results of thermodynamic simulation and is within the composition limit of Cr in 7349 alloy. The alloys were cast in a steel mold as plates of 140 mm thickness. The Cr-modified alloy is herein referred to as 7449+Cr. The chemical compositions measured by optical emission spectrometry (OES) using a Hitachi High-Tech OE750 device are shown in Table 1.

**Figure 1:**
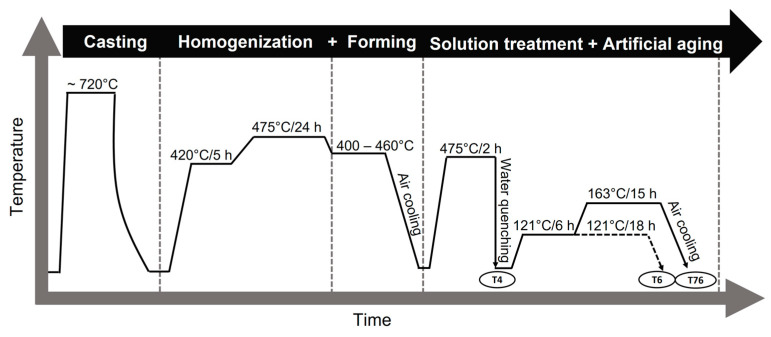
Summary of the processing route for investigated alloys.

The cast plates were homogenized in two steps: 420 °C/5 h + 475 °C/24 h, followed by cooling in air. The homogenized plates were formed by hot forging and finally hot rolled to a final thickness of ~70 mm. To minimize residual stresses, samples with a dimension of ~100 mm × 15 mm × 70 mm were sectioned in the short transverse (ST) direction for solution heat treatment (SHT) and artificial aging. SHT was carried out at 475 °C for 2 h followed by quenching in water. The as-quenched samples were stored at room temperature for a minimum of 3 days to achieve the T4 condition, followed by aging at 121 °C/18 h and 121 °C/6 h + 163 °C/15 h to achieve the T6 and T76 conditions, respectively. A summary of the processing route is shown in Figure 1.

### 2.3. Microstructure Characterization

Samples for the bulk microstructure characterization were mounted in a cold-curing epoxy resin, ground successively using silicon carbide (SiC) paper and then polished with diamond suspension and SiO_2_-based oxide polishing suspension of 0.02 µm particle size at the final polishing step. Samples for grain evaluation in the as-cast condition were etched for ~1.5 min using Barker’s reagent followed by imaging with a ZEISS Axio optical light microscope (OLM) equipped with polarized light. Characterization of the grain structure and recrystallized fraction after processing up to the T4 condition was performed on polished samples by scanning electron microscopy (SEM) using a Zeiss Supra 55 VP (Carl Zeiss Microscopy, Jena, Germany), equipped with an electron backscatter diffraction (EBSD) detector from Oxford Instruments, UK. EBSD measurements were done with a step-size of ~3.5 µm, at an acceleration voltage of 20 kV and a working distance of 19 mm, followed by recrystallized fraction characterization by the grain-orientation spread approach using Aztec Crystal software. Primary phases present in the alloy in the T4 condition were characterized by an SEM equipped with an Oxford energy-dispersive X-ray spectroscopy (EDX) detector.

Microhardness was performed on embedded and polished samples to characterize the age-hardening kinetics of the alloys. Vickers microhardness measurements were performed on a Buehler MICROMET 5104 device using a test force of 0.5 kg (HV 0.5) and an indentation time of 15 s. For each aging condition, an average of 5 different measurements was recorded. 

Dispersoids and grain-boundary precipitate characterization was performed by scanning transmission electron microscopy (S)TEM using a JEOL JEM-F200 equipped with an Oxford energy-dispersive X-ray spectroscopy (EDX) detector. The TEM samples with a diameter of 3 mm were prepared by turning, cutting and grinding, followed by double jet electropolishing with Struers TenuPol 5 in a solution of 25% HNO_3_ and 75% CH_3_OH at a temperature of −30 °C.

### 2.4. Localized Corrosion Test

Pitting corrosion investigation was done by the potentiodynamic polarization (PDP) test using a three-electrode electrochemical cell. The sample was used as the working electrode, saturated calomel electrode as the reference electrode and a platinum wire as the counter electrode. Samples were embedded, ground, polished and masked along the edges to prevent potential crevice corrosion attack. Samples were polarized from −0.3 to 0.3 V with respect to the open circuit potential in an aerated solution of 3.5% NaCl at 25 °C and at a scan rate of 0.5 mV/s by using a Gamry^®^ Reference 600 potentiostat. Prior to polarization, the exposed surfaces were allowed to stabilize at open circuit potential for 1 h.

Samples for the intergranular corrosion (IGC) test had a dimension of 40 × 20 × 2 mm^3^ (L × W × T). They were cut such that the LT-ST plane is exposed to the test solution and IGC penetration is along the longitudinal (L) direction. For each condition, 3 samples were exposed. The test was carried out according to ISO 11846 standard, method B [28]. Samples were immersed for 24 h in the pH ~ 1 solution containing 30 g/L NaCl + 10 mL concentrated HCl at 25 °C. In compliance with the recommended solution volume/specimen area ratio of ≥5 mL/cm^2^, 150 mL of test solution was used for each sample. After 24 h, samples were removed from the solution, rinsed in running water and then with distilled water and dried with warm air. Cross-sections were prepared to reveal corrosion attack in the L direction, followed by embedding, grinding and polishing as described above. Imaging of the penetration depth was done by OLM and an average of 5 deepest attacks was recorded for each sample.

## 3. Results

### 3.1. Phase Diagram and Phase Equilibrium

**Figure 2:**
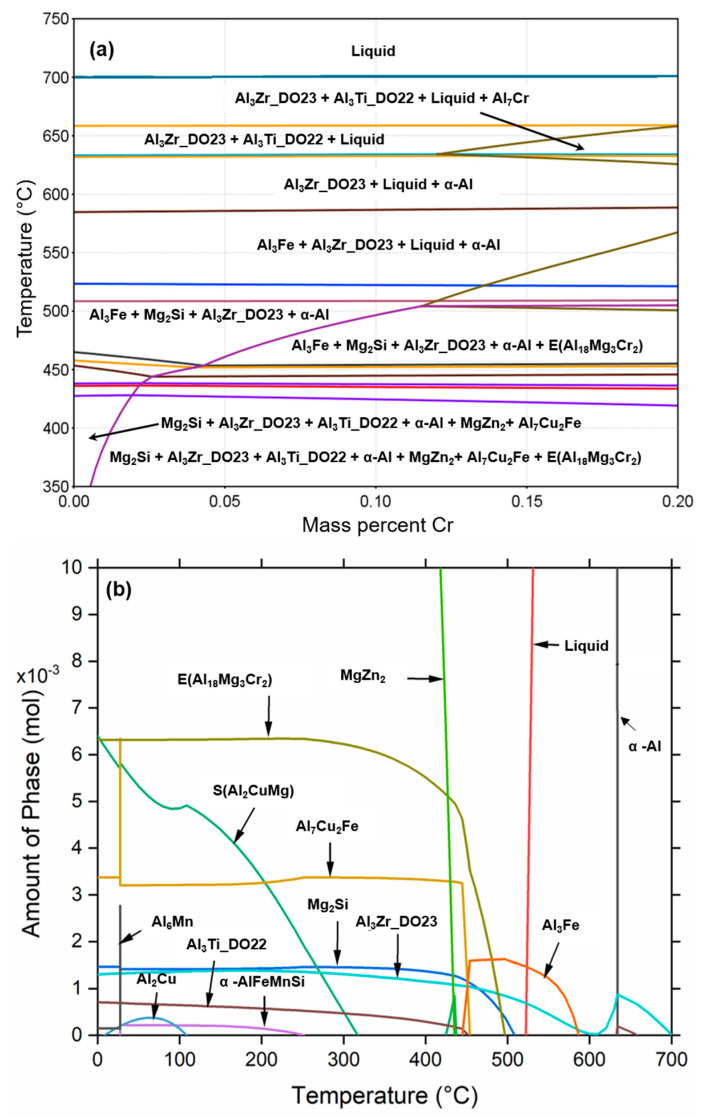
Thermodynamic simulation results: (**a**) phase diagram of 7449 alloy with Cr addition; (**b**) phase equilibrium of 7449 + 0.1 wt.% Cr replotted with OriginPro.

The phase diagram and phase equilibrium of the investigated 7449 alloy composition with additional Cr addition are shown in Figure 2. The phase diagram shown in Figure 2a indicates the formation of a Cr-rich primary phase with Al_7_Cr stoichiometry at a Cr content of approximately ≥0.12 wt.%. Al_7_Cr has been reported to be detrimental to mechanical properties [29,30]. In addition, the formation of E (Al_18_Mg_3_Cr_2_) dispersoids occurs with Cr content as low as 0.01 wt.%. However, at such low concentration, the dispersoids are only distributed in colonies [13]. Therefore, to ensure the formation and uniform distribution of E (Al_18_Mg_3_Cr_2_) dispersoids, minimize quench sensitivity and hinder Al_7_Cr formation, a Cr content of 0.1 wt.% is considered optimum and was chosen in this study. The phase equilibrium diagram of 7449 with 0.1 wt.% Cr shown in Figure 2b confirms the formation of only E (Al_18_Mg_3_Cr_2_) dispersoids.

### 3.2. Microstructure Evolution

#### 3.2.1. Grain Structure

**Figure 3:**
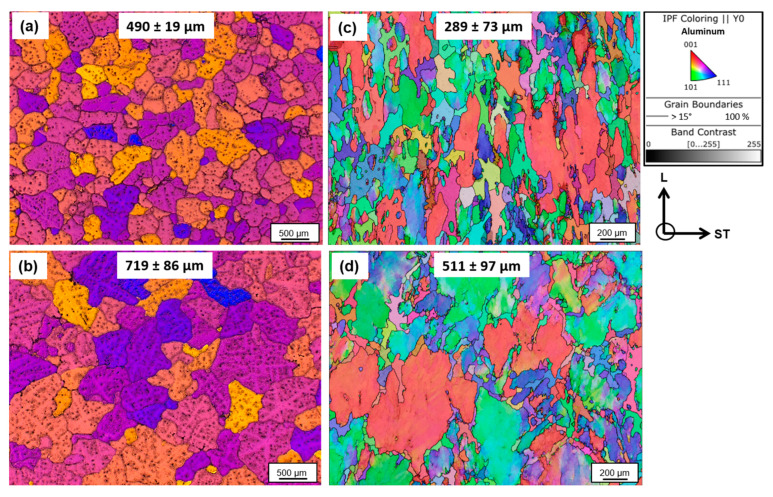
Barker-etched images of grains in as-cast condition: (**a**) 7449; (**b**) 7449+Cr and EBSD IPF maps after processing to T4 state; (**c**) 7449; (**d**) 7449+Cr.

The results of the grain structure of the alloys after casting and after processing to T4 are shown in Figure 3. Barker-etched images of the alloys in as-cast condition in Figure 3a,b show the formation of equiaxed grain structure in both alloys with bimodal grain size. However, an obvious influence of Cr addition on the as-cast grain size is evident, which was found to be ~1.5 times larger than the base alloy. The corresponding EBSD inverse pole figure (IPF) maps of the alloys along the L-ST plane, after processing to the T4 condition, are shown in Figure 3c,d. The presence of large deformed grains, slightly oriented along the longitudinal direction, and small recrystallized grains can be seen in both alloys. A weighted average grain size (equivalent circle diameter) of 511 ± 97 was measured for 7449+Cr, which was ~1.8 times larger than 289 ± 73 measured for 7449 alloy.

#### 3.2.2. SEM Characterization of Primary Phase and Recrystallized Fraction

**Figure 4:**
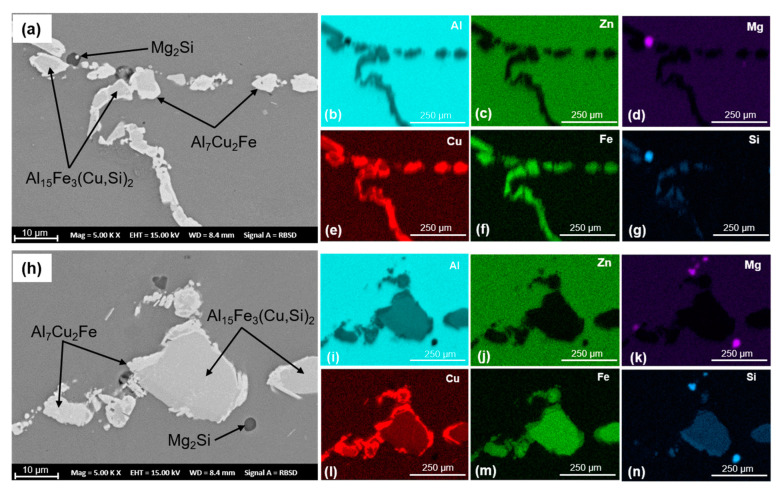
Coarse primary phases present in the alloys after processing to T4 state and the corresponding EDX maps for 7449 (**a**–**g**) and 7449+Cr (**h**–**n**).

The coarse primary phases present in the alloys after solution heat treatment and corresponding EDX maps are shown in Figure 4. The phases identified by EDX had compositions close to Al_7_Cu_2_Fe, Al_15_Fe_3_(Cu,Si)_2_ and Mg_2_Si. No coarse Al_7_Cr phase was identified in 7449+Cr alloy. A comparable coarse primary phase area fraction of 8.5 ± 0.3% and 9.1 ± 1.7% were measured for 7449 and 7449+Cr, respectively. Therefore, the effect of varying amount of coarse primary phase on dynamic recrystallization, which occurs by particle stimulated nucleation (PSN) in 7xxx alloys, can be ruled out. 

**Figure 5:**
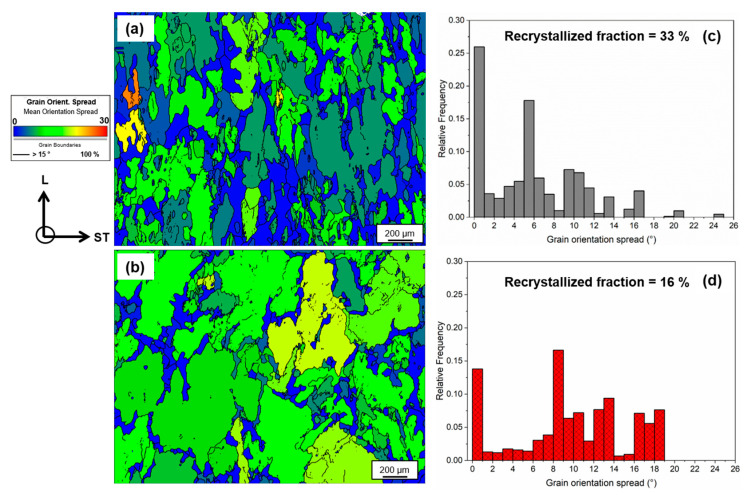
GOS maps of the alloys in T4 state: (**a**) 7449 and (**b**) 7449+Cr. The corresponding relative frequency distributions are shown for (**c**) 7449 and (**d**) 7449+Cr.

**Figure 6:**
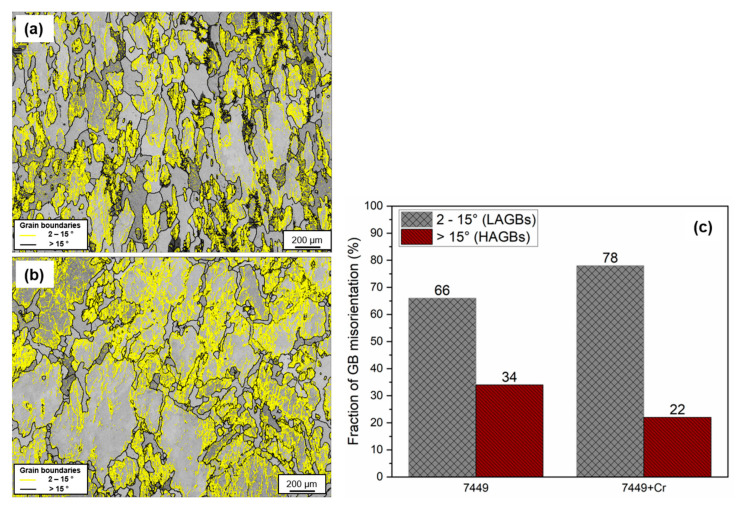
Grain-boundary misorientation in T4 state: (**a**) 7449 and (**b**) 7449+Cr. (**c**) Fraction of LAGBs and HAGBs.

The grain-orientation spread (GOS) maps of both alloys, with high-angle grain boundaries (misorientation > 15°) in black, are shown Figure 5a,b. GOS measures the average misorientation between each pixel within a grain, with GOS of recrystallized grains being ≤3°, while deformed grains have a GOS of >3° [31,32]. The corresponding relative frequency distribution plots shown in Figure 5c,d suggest the presence of recrystallized and deformed grains. Using a threshold of 3°, a recrystallized fraction of 16% was determined for 7449+Cr, while the 7449 alloy had a higher recrystallized fraction of 33%. The grain-boundary misorientation distribution showing low-angle grain boundaries (LAGBs) with misorientation angle ranging between 2–15° and high-angle grain boundaries (HAGBs) with misorientation angle > 15° are shown in Figure 6. With Cr addition, the fraction of HAGBs decreased from 34% to 22%.

#### 3.2.3. TEM Microstructure Characterization

**Figure 7:**
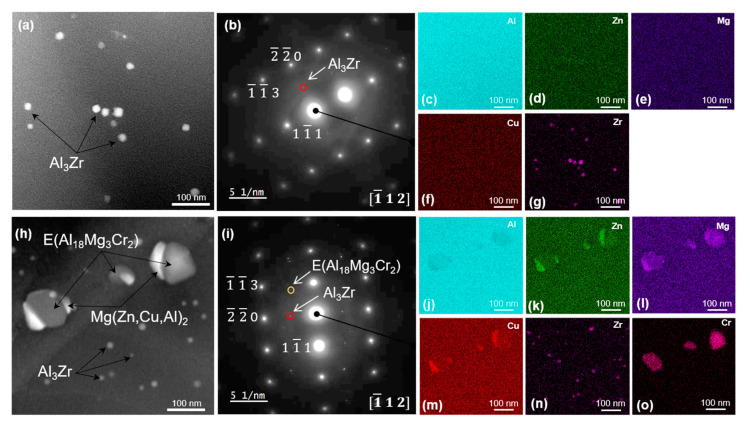
STEM dark-field images and SAED patterns of 7449 (**a**,**b**) and 7449+Cr (**h**,**i**). The corresponding EDX maps of STEM images in (**a**) and (**h**) for 7449 and 7449+Cr, respectively, are shown in (**c**–**g**) and 7449+Cr (**j**–**o**).

STEM dark-field images, selected area electron diffraction (SAED) patterns and EDX maps of the matrix microstructure of both alloys in the T4 condition showing the distribution of dispersoids, are shown in Figure 7. The presence of Zr-rich particles with a size of 15–30 nm, which are Al_3_Zr dispersoids, are evident in both alloys. The SAED patterns in Figure 7b,i taken along the ${\left[\;\bar{1}\;1\;2\;\right]}_{Al}$ zone axis show diffraction spots of Al_3_Zr dispersoids at ½ {2 2 0}_Al_ position [33]. In addition, Cr-rich E (Al_18_Mg_3_Cr_2_) dispersoids can be seen in the matrix of 7449+Cr in Figure 7h. Compared to the Al_3_Zr dispersoids, the E (Al_18_Mg_3_Cr_2_) dispersoids are larger, with a size of ~ 70–150 nm and with evidence of heterogeneous nucleation of *η* Mg(Zn,Cu,Al)_2_ phase. The electron diffraction pattern of 7449+Cr in Figure 7i shows additional diffraction spots relative to that of 7449 in Figure 7b. The E (Al_18_Mg_3_Cr_2_) was oriented parallel to the {2 0 1}_Al_ plane in agreement with previous study [17]. The presence of an additional type of dispersoid with Cr addition indicates a difference in the dispersoid volume fraction in both alloys, which is higher in 7449+Cr. The E (Al_18_Mg_3_Cr_2_) dispersoids are formed in Cr-containing 7xxx alloys during homogenization heat treatment for the purpose of recrystallization inhibition. The presence of a higher volume fraction of dispersoids in 7449+Cr contributed to the observed decrease in recrystallized fraction in the alloy, compared with the 7449 alloy.

**Figure 8:**
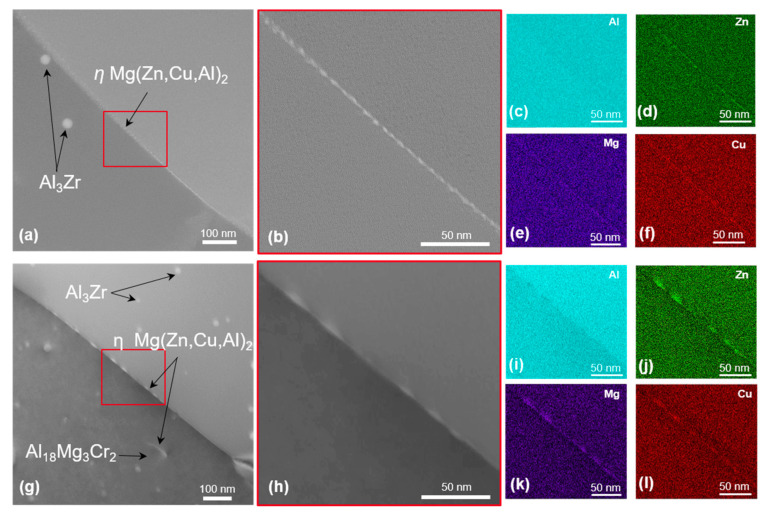
STEM dark-field images and EDX maps of alloys in T4 state: (**a**–**f**) 7449 and (**g**–**l**) 7449+Cr. The red boxes indicate the region of interest for EDX mapping.

**Figure 9:**
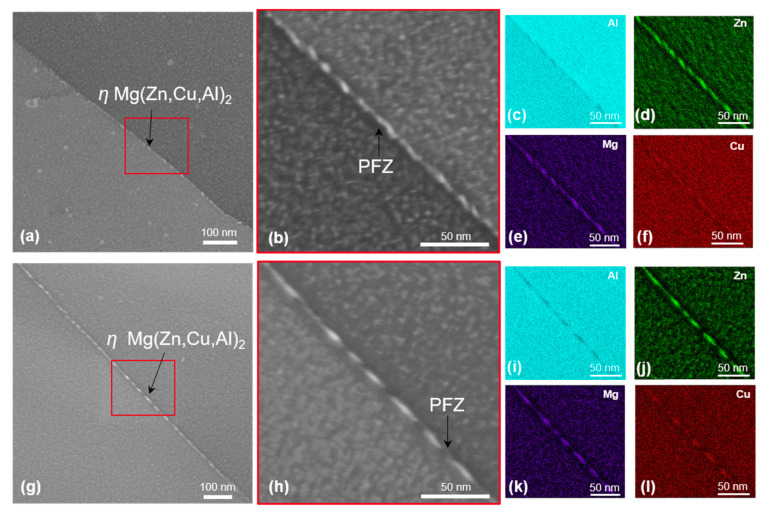
STEM dark-field images and EDX maps of alloys in T6 state: (**a**–**f**) 7449 and (**g**–**l**) 7449+Cr. The red boxes indicate the region of interest for EDX mapping.

**Figure 10:**
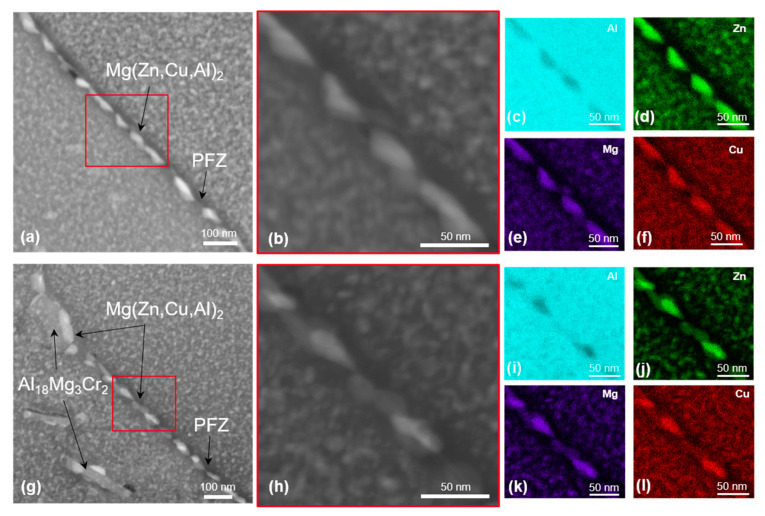
STEM dark-field images and EDX maps of alloys in T76 state: (**a**–**f**) 7449 and (**g**–**l**) 7449+Cr. The red boxes indicate the region of interest for EDX mapping.

In addition, the EDX maps of 7449+Cr shows the presence of Cu and Zn in the E (Al_18_Mg_3_Cr_2_) dispersoids, indicating a deviation from the known stoichiometry of this phase. STEM dark-field images and corresponding EDX element maps of grain-boundary microstructure in T4, T6 and T76 conditions are shown in Figure 8, Figure 9 and Figure 10, respectively. In the T4 condition shown in Figure 8, the grain boundaries of both alloys are covered with fine particles, which are believed to have formed during quenching. The corresponding EDX maps of 7449 and 7449+Cr in Figure 8c–f and i–l, respectively, suggest that these quench-induced particles are likely the *η* Mg(Zn,Cu,Al)_2_ phase. Furthermore, it can be seen that the quench-induced *η* Mg(Zn,Cu,Al)_2_ phase are slightly larger and discreetly populate the grain boundary of 7449+Cr compared to that of 7449 alloy. Furthermore, segregation of solute atoms at the grain boundaries in this aging condition has been reported by several investigations [34,35,36] and therefore cannot be ruled out in this study. Figure 9 shows the STEM grain-boundary microstructure of the alloys in the T6 condition. Enhanced matrix precipitation of what is believed to be mainly the metastable *η’* strengthening phase was observed in both alloys. Compared to the T4 condition, an increase in the size of the grain boundary *η* Mg(Zn,Cu,Al)_2_ phase was equally observed, with a narrow precipitate-free zone (PFZ). However, similar to the observation in the T4, the *η* Mg(Zn,Cu,Al)_2_ phases at the grain boundary of 7449+Cr are larger with higher interparticle spacing compared with that of 7449 alloy. In the T76 condition, shown in Figure 10, coarsening of the matrix precipitates and the grain boundary *η* Mg(Zn,Cu,Al)_2_ phase compared to the T4 and T6 conditions was observed in both alloys. In addition, the formation of a wider PFZ is clearly visible in this condition. Only a minor difference in the grain-boundary coverage of both alloys was observed in the T76 condition, which was also lower in 7449+Cr. However, the largest interparticle spacing occurred in the T76 condition relative to the T4 and T6 conditions.

The increase in the size of matrix and grain-boundary precipitates from T4 to T6 and to T76 conditions is due to enhanced diffusion of solute atoms, particularly Zn and Mg, with increasing aging temperature and time. Recall that the T76 aging was done in two stages, with the second stage being at a higher temperature, where the atomic diffusion rate is certainly high relative to the temperature of the first stage and the T6 aging [1]. In addition, the Cu-content of grain-boundary precipitates increases with increasing aging temperature, owing to the low diffusion coefficient of Cu compared to that of Zn and Mg [37,38,39,40]. Hence, it can be assumed that the Cu content of the grain boundary *η* Mg(Zn,Cu,Al)_2_ phase increases in the following order; T4 < T6 < T76.

#### 3.2.4. Hardness Evolution

**Figure 11:**
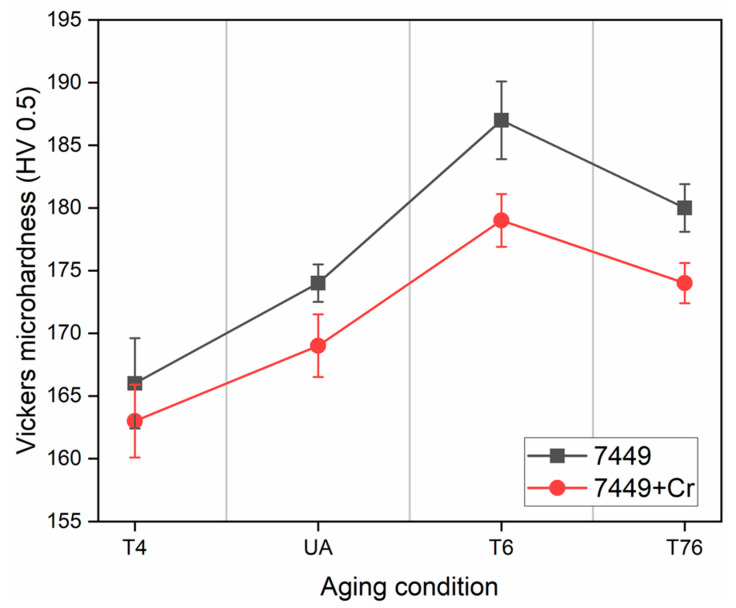
Microhardness test results showing the effect of minor Cr addition on hardness evolution.

The hardness evolution of the alloys is shown in Figure 11. In the T4 condition, 7449+Cr showed a 2% lower hardness relative to the Cr-free alloy. This slight decrease in hardness is attributable to the presence of E (Al_18_Mg_3_Cr_2_) dispersoids and large quench-induced *η* Mg(Zn,Cu,Al)_2_ phase. During artificial aging to the under-aged UA (121 °C/3 h), peak-aged T6 and over-aged T76 conditions, the hardness of the Cr-modified 7449+Cr alloy remained inferior to that of the base alloy. However, only a maximum of 4% hardness drop was observed with Cr addition in T6.

The known precipitation sequence from supersaturated solid solution (SSSS) in 7xxx alloys is SSSS—Guinier–Preston (GP) zones—metastable *η’* phase—equilibrium *η* phase [1,41]. The strengthening mechanisms are known to be due to coherence strain fields in T4 and under-aged conditions. On the other hand, a mechanism change to mainly particle shearing due to the formation of semicoherent *η’* in the T6 condition results in hardness increase [41,42]. Furthermore, coarsening of the semicoherent *η’* and/or its transformation to the incoherent equilibrium *η* Mg(Zn,Cu,Al)*_2_* in the T76 condition results a in gradual mechanism change to Orowan looping, leading to a drop in hardness [1]. In 7449+Cr, the additional E (Al_18_Mg_3_Cr_2_)/*η* Mg(Zn,Cu,Al)_2_ phase results in alloying element depletion, which lowers the precipitate volume fraction and therefore results in the overall hardness drop relative to 7449.

### 3.3. Pitting Corrosion Susceptibility

**Figure 12:**
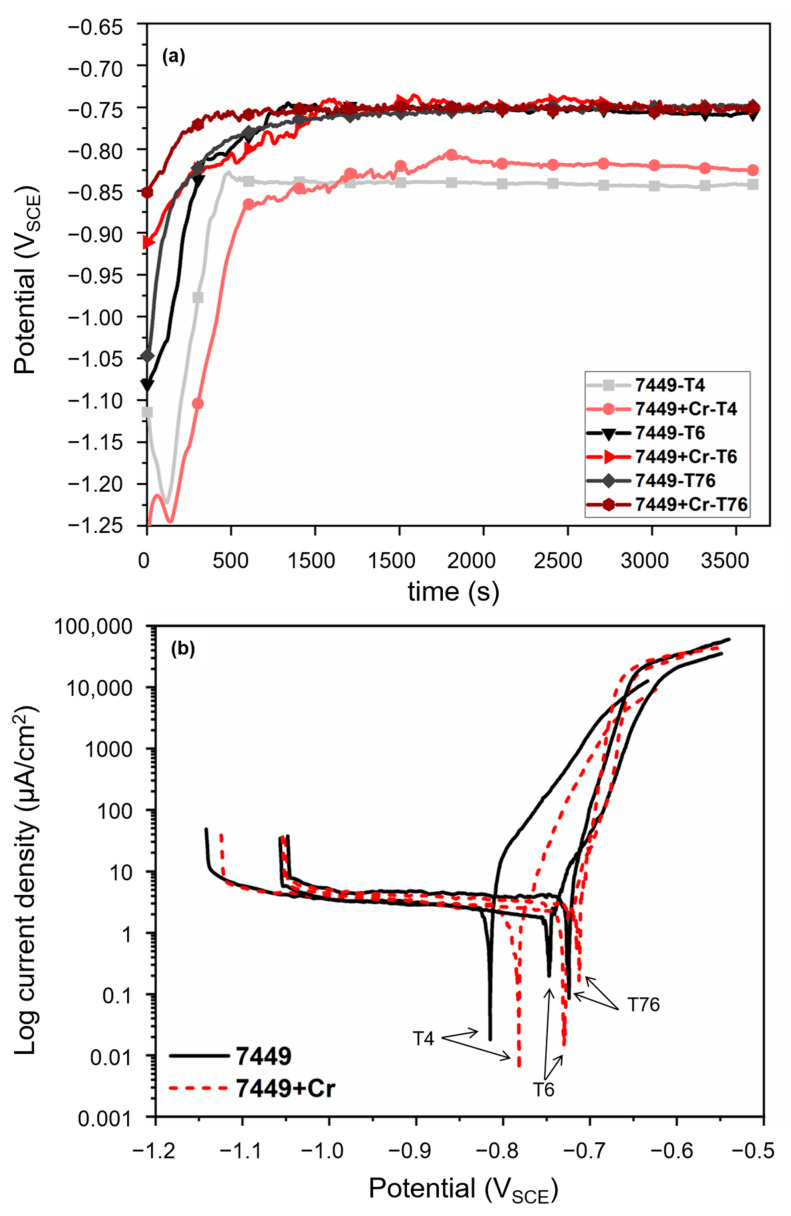
Initial open circuit delay curves (**a**) and potentiodynamic polarization curves (**b**) of alloys in aerated 3.5% NaCl solution at 25 °C.

The initial open circuit delay and the corresponding potentiodynamic polarization (PDP) curves of the alloys in different heat-treatment conditions are shown in Figure 12. The corrosion potential curves in Figure 12a show potential stabilization during initial delay prior to polarization. The PDP curves in Figure 12b show that in the T4 state, a higher open circuit potential (OCP) was measured in 7449+Cr than in 7449. Artificial aging resulted in a shift of the OCP to a more positive direction. This indicates that the removal of alloying elements, particularly Zn and Mg due to precipitation of metastable *η’* and equilibrium *η* Mg(Zn,Cu,Al)_2_ phase, shifts the electrochemical potential of the bulk matrix towards the noble direction. Similar to the T4 state, a slightly higher OCP was observed in 7449+Cr alloy in both T6 and T76 states. A minor shift in the cathodic branch to higher current densities was also observed, particularly in the T76 state. Anodic polarization resulted in a rapid increase in the current density from the OCP, indicating a local breakdown at the OCP or close to it. Therefore, it can be assumed that the breakdown potential (E_b_) is equal to the OCP. Although the presence of different breakdown potentials in Cu-containing 7xxx alloys have been reported by previous investigations [35,37,38,40,43], no strong evidence of a second breakdown potential was observed in this study. This might be due to differences in alloy chemistry, sample surface, test solution conditions and the potential scan rate.

**Figure 13:**
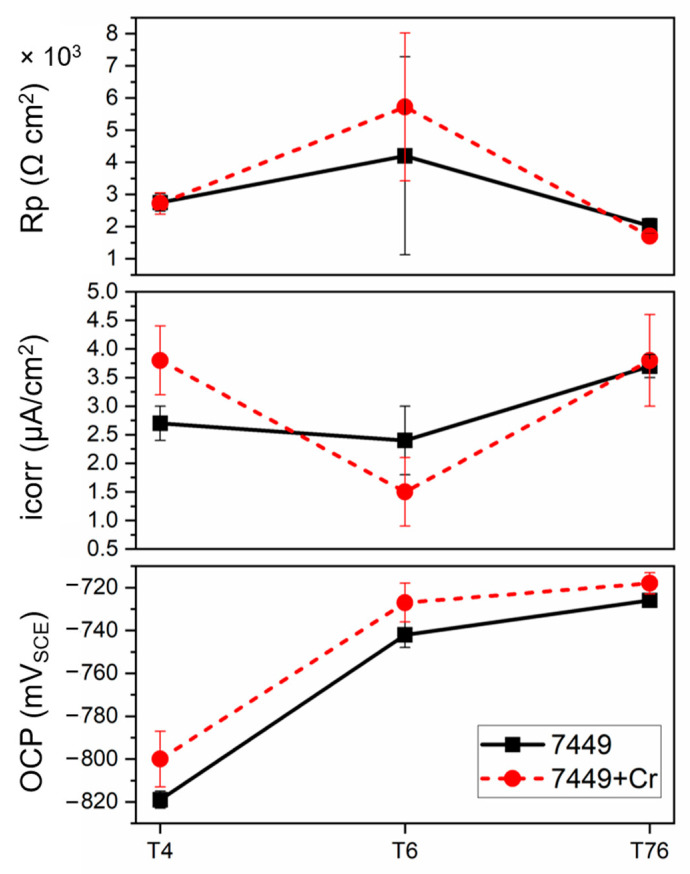
Measured electrochemical parameters from potentiodynamic polarization tests in different aging conditions.

The parameters estimated from the polarization measurements are presented in Figure 13. Despite a clear increase in the breakdown potential with Cr addition and with artificial aging, the measured current density at OCP (i_corr_) and the calculated polarization resistance (R_p_) did not suggest a decrease in corrosion susceptibility. However, since corrosion of 7xxx alloys in NaCl solution is more localized than uniform, the measured i_corr_ may have been affected by localized corrosion and therefore inadequate for estimating corrosion rate of the alloys during PDP. The i_corr_ first decreased after T6 aging and then increased in the T76 condition. This was in agreement with the calculated average R_p_, which increased in the T6 condition, followed by a decrease in the T76 state. In the T4 condition, a higher i_corr_ was measured in 7449+Cr compared to 7449. Nevertheless, a comparable R_p_ was calculated in both alloys in the T4 state. An opposite behavior was observed in the T6 state, with 7449 having a higher i_corr_ and a lower R_p_ than 7449+Cr, while in the T76 state, a comparable i_corr_ and R_p_ was determined. These differences in corrosion susceptibility are correlated to the microstructure and presented in the discussion section.

### 3.4. Intergranular Corrosion

**Figure 14:**
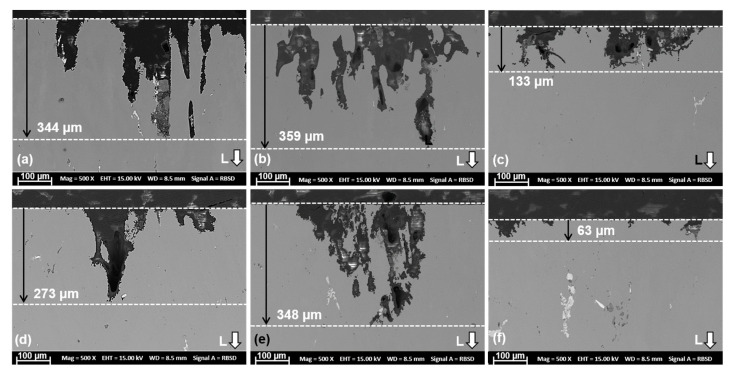
SEM images showing corrosion attack after the IGC test: (**a**) 7449-T4, (**b**) 7449-T6, (**c**) 7449-T76, (**d**) 7449+Cr-T4, (**e**) 7449+Cr-T6, (**f**) 7449+Cr-T76.

Cross-sections of the samples after the intergranular corrosion (IGC) test in pH 1 solution are shown in Figure 14. 

**Figure 15:**
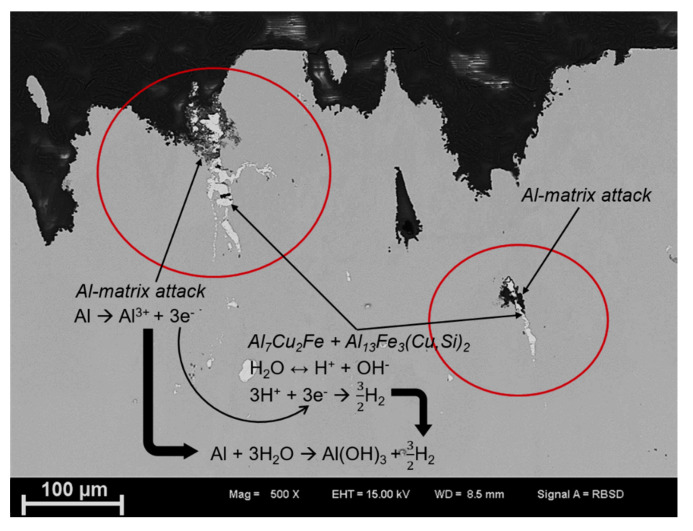
SEM image showing evidence of matrix attack in the vicinity of AlCuFe particles.

The corrosion behavior in T4 and T6 conditions show severe attack compared to the T76 condition. The morphology of the attack appears to be a combination grain boundary and adjacent matrix attack, mainly along the longitudinal direction. However, the corrosion kinetics differs with aging condition and with Cr addition due to differences in microstructure and grain-boundary microchemistry. Furthermore, local attack in the vicinity of AlCuFe (Al_7_Cu_2_Fe + Al_13_Fe_3_(Cu,Si)_2_) intermetallic particles, as shown in Figure 15, was observed in both alloys and in all the aging conditions. It is therefore plausible that the local breakdown of the protective oxide layer and corrosion initiation occurred in the vicinity of these particles according to the presented reactions, due to differences in electrochemical potential of the intermetallics and the matrix.

**Figure 16:**
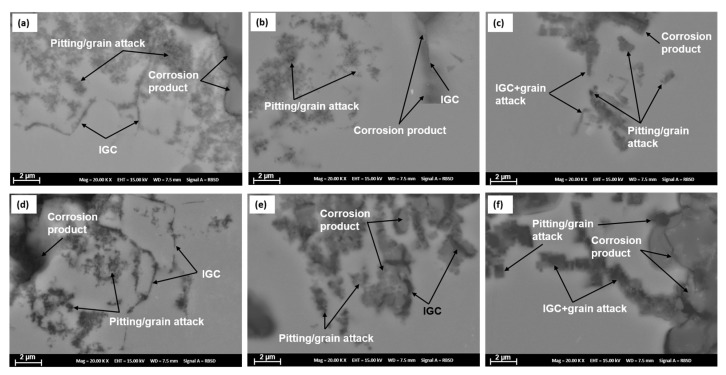
SEM images of the corrosion front showing observed corrosion mechanisms: (**a**) 7449-T4, (**b**) 7449-T6, (**c**) 7449-T76, (**d**) 7449+Cr-T4, (**e**) 7449+Cr-T6, (**f**) 7449+Cr-T76.

SEM images of the corrosion front of the alloys in Figure 16 confirms IGC, pitting and grain attack as the corrosion mechanisms in T4, T6 and T76 aging, suggesting no change in corrosion mechanisms with Cr addition and with heat treatment. 

**Figure 17:**
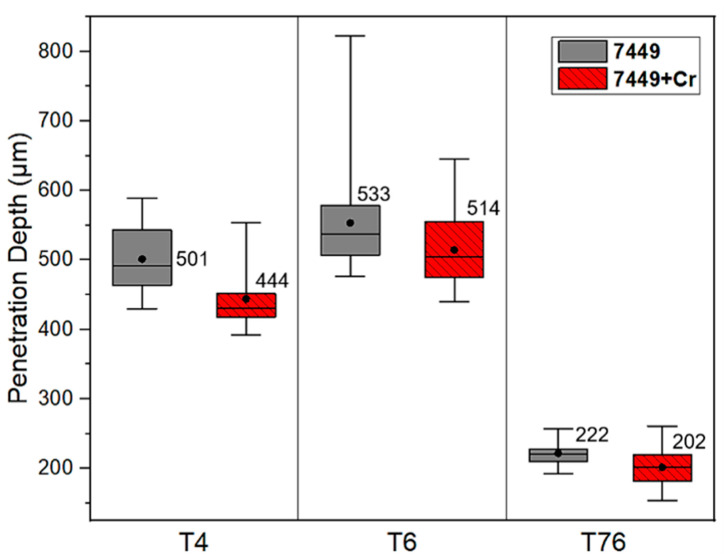
Box plots of measured corrosion penetration depth after 24 h immersion in a pH 1 IGC solution. The black circles and values indicate the average penetration depth.

However, the corrosion penetration depth measured in all the investigated samples and the different aging conditions, shown in Figure 17, indicates differences in the average penetration depth. The average penetration depth (indicated by black circles and values) with respect to temper conditions increased as follows: T76 < T4 < T6, indicating the highest attack in the T6 condition. Nevertheless, 7449+Cr showed a lower average depth of attack than 7449 in all the studied aging conditions, indicating a decrease in corrosion susceptibility with Cr addition. This decrease in corrosion susceptibility was, however, less obvious after prolonged aging in the T76 condition.

## 4. Discussion

### 4.1. Influence of Cr on Microstructure

The results of this study indicate that 0.1 wt.% Cr addition to an Al-Zn-Mg-Cu-Zr alloy has an influence on the microstructural features, such as grain size, recrystallized fraction, hardness evolution and grain-boundary precipitation. A trace amount of Ti is added in 7xxx alloys to form Al_3_Ti particles, which act as heterogeneous nucleation sites for α-Al by peritectic reaction during solidification, resulting in the formation of fine equiaxed grains [14]. Although transition metals such as Zr and Cr with low solid solubility in Al equally exhibit a grain-refinement effect in pure Al [44,45,46], they are mainly added in 7xxx alloys to inhibit recrystallization and grain growth during post-casting processing and to reduce their susceptibility to stress corrosion cracking [13,14,47]. However, in commercial alloys with Fe and Si impurities, Cr and Zr exhibit a poisoning effect on the Al_3_Ti, suppressing its grain-refinement ability [45,48,49]. 

The mechanism of heterogeneous nucleation kinetic is governed by the equilibrium of interfacial energies and contact angle according to the equation below [46]:

(1) Cos θ=(γlp− γsp)γls

where θ = contact angle, $\gamma_{\mathrm{lp}}$ = liquid/particle interface energy, $\gamma_{\mathrm{sp}}$ = solid/particle interface energy and $\gamma_{\mathrm{ls}}$ = liquid/solid interface energy. The interface energies necessary to lower the contact angle to ensure adequate wetting of the nucleating crystals is strongly affected by the alloying elements [46,49]. According to Arjuna Rao et al. [45], the poisoning effect of Cr is not only affected by the amount of Cr present, but also on the level of Fe and Si impurities. The interaction of these elements with Al_3_Ti particles form complex aluminides with poor heterogeneous nucleation properties, due to unfavorable alteration of the interface energies [46]. This explains the result of larger grain size observed in the as-cast microstructure (Figure 3) of 7449+Cr alloy, compared with the base alloy.

The type, size and volume fraction of dispersoids present during forming and solution heat treatment, affects the degree of recrystallization. Fine coherent Al_3_Zr dispersoids in Al-Zn-Mg-Cu-Zr alloys are effective in inhibiting recrystallization, which occurs by particle stimulated nucleation (PSN) and grain growth during thermomechanical processing [6,50]. Cr, on the other hand, forms coarse incoherent E (Al_18_Mg_3_Cr_2_) dispersoids, with less recrystallization inhibition properties and higher quench-sensitivity effect compared with Al_3_Zr [12,13,51]. The mechanism of recrystallization inhibition by dispersoids is defined by the amount of Zener pinning force (P_z_), defined below [6]:

(2) PZ =(CVfγ)r

where $V_{f}$ = volume fraction of dispersoids, γ = grain-boundary energy, r = radius of dispersoid and C is a constant. For effective recrystallization inhibition, P_z_ should be equal to or greater than the driving force for recrystallization (stored energy accumulation at coarse intermetallic particles) [6,7]. Equation (2) indicates that a higher volume fraction of dispersoids and a smaller dispersoid radius favor recrystallization inhibition. Hence, relative to Zr alone, combined addition of Zr and Cr in 7xxx alloys ensures better recrystallization inhibition. In addition, recrystallization is enhanced by an increase in the degree of deformation and as-cast grain refinement, due to a large amount of stored energy [52]. Therefore, a lower driving force for recrystallized grain nucleation is expected in 7449+Cr compared to 7449 due to the larger as-cast grain size observed with 0.1 wt.% Cr addition. 

The degree of recrystallization affects the grain-boundary misorientation and grain-boundary precipitation during subsequent artificial aging. Unrecrystallized/partially recrystallized microstructures are dominated by recovered substructure with low-angle grain boundaries (LAGBs). During recrystallization, subgrain migration and grain growth occurs, resulting in an increase in the fraction of high-angle grain boundaries (HAGBs) [53,54,55], which is in agreement with the result of the grain-boundary misorientation distribution shown in Figure 6. The high interface energy of HAGBs makes them prone to enhanced grain-boundary precipitation and precipitate free zone (PFZ) formation, compared to LAGBs [22,54]. The higher fraction of HAGBs in 7449 relative to 7449+Cr may have contributed to a higher amount of grain boundaries with enhanced precipitation in the studied conditions, as shown by the STEM results in Figure 8, Figure 9 and Figure 10.

### 4.2. Quench Sensitivity Effect of Cr 

High quench sensitivity remains a major drawback of Cr as a recrystallization inhibition element in 7xxx alloys, particularly in thick plates where thermal gradients during quenching from solution heat treatment are inevitable [56]. This is shown in the STEM matrix microstructure characterization results in the T4 condition, shown in Figure 7, where nucleation of *η* Mg(Zn,Cu,Al)_2_ at the E (Al_18_Mg_3_Cr_2_)/Al-matrix interface was observed. The age hardening curves in Figure 11 confirm this phenomenon, with lower microhardness values measured in 7449+Cr relative to 7449. Large interface energy exists between E (Al_18_Mg_3_Cr_2_) dispersoids and Al-matrix due to their large size, incoherence, crystal structure and orientation relationship with the Al-matrix, making them prone to heterogeneous precipitation during quenching [16,17,57]. Such heterogeneous nucleation reduces the concentration of vacancies and alloying elements needed to form strengthening precipitates, and is therefore undesirable. According to Figure 7 and also confirmed by previous investigations [17,58], E (Al_18_Mg_3_Cr_2_) dispersoids contain minor amounts of Zn and Cu, which further indicates a reduced amount of alloying elements in the matrix of 7449+Cr. Lowering the Cr content could potentially minimizes quench sensitivity of 7xxx alloys [13,59], but this will be at the detriment of recrystallization inhibition due to decrease in the dispersoids volume fraction. The present study shows about 50% decrease in recrystallized fraction with 0.1 wt.% Cr addition in 7449, which is quite significant considering that only a maximum of 4% drop in hardness was observed after artificial aging to the T6 condition.

### 4.3. Localized Corrosion Initiation and Propagation

Initiation of localized corrosion starts with the local breakdown of the protective oxide layer in a chloride-containing environment, followed by the galvanic coupling between cathodic and anodic microstructural features, such as coarse cathodic intermetallic phases, grain-boundary precipitates, PFZ and the matrix [34]. In 7xxx alloys and also observed in this study (Figure 15), localized corrosion initiates mainly in the vicinity of large micron-sized intermetallics, such as Al_7_Cu_2_Fe, Al_2_Cu, Al_3_Fe and Al_15_Fe_3_(Cu, Si)_2_ due their more noble potentials relative the matrix [9,10,34,40]. According to Birbilis and Buchheit [10], coarse noble particles have high electrochemical activity and therefore sustain large cathodic currents which drive the pitting process. On the contrary, nanosized noble particles such as Al_3_Zr dispersoids have no adverse effect on corrosion kinetics, due to their inability to sustain such large cathodic currents. At the time of this investigation, no information on the electrochemical potential of E (Al_18_Mg_3_Cr_2_) dispersoid was found in the literature. Therefore, the precise role of this phase on the breakdown potential is still unknown and is beyond the scope of this work. The single breakdown potential which occurred at OCP is related to the amount of active elements, particularly Mg and Zn in solid solution [34,35]. During artificial aging to the T6 condition, precipitation of Mg- and Zn-rich *η’* strengthening particles occur as indicated by STEM microstructure characterization and hardness measurements, resulting in a shift in the OCP towards a more positive direction. This shift towards a more positive potential is enhanced in the T76 condition due to precipitate growth and transformation to the equilibrium *η* Mg(Zn,Cu,Al)_2_ phase. Formation of E (Al_18_Mg_3_Cr_2_) dispersoids in 7449+Cr, which contains minor amounts of Zn and Cu, followed by the formation of quench-induced *η* Mg(Zn,Cu,Al)_2_ phase at the E (Al_18_Mg_3_Cr_2_)/Al-matrix interface ultimately depletes the amount of Zn, Mg and Cu in solid solution. Therefore, it can be concluded that the more positive OCP in 7449+Cr is related the presence of E (Al_18_Mg_3_Cr_2_) dispersoids and the quench-induced *η* Mg(Zn,Cu,Al)_2_ phase associated with this phase.

During corrosion tests, dissolution of *η* Mg(Zn,Cu,Al)_2_ phase in 7xxx alloys occurs due to its high reactivity and therefore affects the electrochemical behavior of 7xxx alloys [10]. Such selective anodic dissolution is believed to be responsible for an increase in the cathodic activity, as observed by the shift in the cathodic branch of the PDP curves towards higher current densities. This is also indicated by the higher i_corr_ measured in the T4 condition of 7449+Cr, where a higher amount of *η* Mg(Zn,Cu,Al)_2_ phase is present relative to 7449, due to precipitation at E(Al_18_Mg_3_Cr_2_)/Al-matrix interface. Wloka and Virtanen [60] reported the occurrence of current transients in the passive range of AA7010-T6 alloy during polarization measurements and attributed it to the dissolution of *η* phase. This selective dissolution is believed to have an influence on the measured i_corr_ in the T6 and T76 conditions. Recall that 7449 showed ~4% higher hardness relative to 7449+Cr in the T6 condition, indicating a higher volume fraction of strengthening *η’* phase, which could be the reason for the higher i_corr_ and lower R_p_ measured in 7449-T6 compared to 7449+Cr-T6. This, however, does not explain the comparable i_corr_ and R_p_ measured in both alloys in the T76 condition, where only about 3% hardness drop was observed in 7449+Cr relative to 7449.

Similar to short-term polarization measurements, the corrosion mechanism associated with longer duration (24 h) immersion tests, like the IGC test, is equally related to the grain-boundary microstructure and microchemistry, which varies depending on alloy chemistry and aging condition, as shown in this study. The presence of continuous anodic grain-boundary precipitates affects IGC susceptibility in both T4 and T6 conditions. In the T76 condition, the presence of coarse discontinuous grain-boundary precipitates with a higher Cu content relative to the T4 and T6 conditions lowers the dissolution rate of the grain boundaries. Ramgopal et al. [40] reported that the grain-boundary precipitates contain 2.5 times more Cu in the T7 condition compared to the T6 condition, indicating a significant difference in the microchemistry of grain-boundary precipitates with a temper condition. Therefore, the increase in size and Cu content of grain-boundary precipitates lowers their dissolution rate in the T76 condition. Additionally, Figure 9 and Figure 10 show a larger PFZ width in T76 compared to the T6 condition. The dissolution kinetic of the PFZ is controlled by the microchemistry of the grain-boundary precipitates, especially the Cu content [40]. Dissolution of the grain-boundary precipitates in the T76 condition results in high local concentration of Cu ions at the grain-boundary region, which ennobles the PFZ in this condition compared to the T6 condition [40]. However, with longer immersion time, IGC occurs in the T76 condition, but at a lower rate, in comparison to the T4 and T6 conditions. 

**Figure 18:**
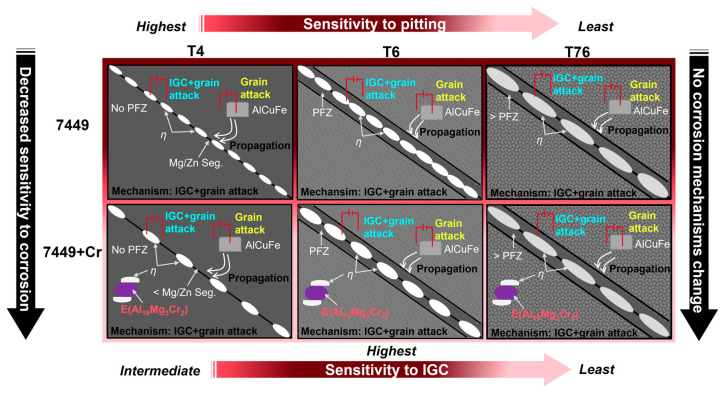
Schematic diagram showing the role of minor Cr addition on microstructure and its correlation to the observed corrosion mechanisms.

Correlation of the microstructure and IGC behavior of both alloys indicates that by lowering the fraction of HAGBs to LAGBs through recrystallization inhibition, e.g., by Cr and/or rare earth element addition, GB precipitation becomes less favorable, and a less continuous corrosion path can be achieved [19,20,22,54]. In addition, the amount of Mg, Zn and Cu diffusing or segregating at the grain boundaries is expected to be less in 7449+Cr alloy due to the depletion of alloying elements resulting from the formation of E (Al_18_Mg_3_Cr_2_) dispersoids and quench-induced *η* Mg(Zn,Cu,Al)_2_ at its interface with the matrix, thereby limiting the otherwise favorable grain-boundary precipitation. These observations are believed to be responsible for the decreased susceptibility to localized corrosion with 0.1 wt.% Cr addition in a Zr-containing 7xxx alloy. Based on the findings of this investigation, a schematic model of the role of Cr addition on microstructure and its correlation to localized corrosion susceptibility is presented in Figure 18.

## 5. Conclusions

The role of 0.1 wt.% Cr addition on the microstructure evolution and localized corrosion susceptibility of a technical Al-Zn-Mg-Cu-Zr alloy, with no rare earth element, has been systematically investigated. The following can be concluded:

1. Under equilibrium conditions and within the studied composition range, formation of a coarse Al_7_Cr phase does not occur in 7449 alloy with Cr content of ≤0.12 wt.%
2. Addition of 0.1 wt.% Cr in 7449 alloy results in the coarsening of the as-cast grain size and in the formation of Cu- and Zn-containing E (Al_18_Mg_3_Cr_2_) dispersoids, increasing the dispersoid volume fraction. These result in an improved recrystallization inhibition during processing.
3. Quench sensitivity occurs with 0.1 wt.% Cr addition in 7449 alloy, which affects the hardness evolution. However, only a maximum of 4% drop in hardness was observed in peak-aged condition.
4. Addition of 0.1 wt.% Cr shifts the OCP/breakdown potential (E_b_) to a more positive direction after T4, T6 and T76 aging due to depletion of alloying elements, particularly Mg and Zn, from solid solution due to the formation of Cu- and Zn-containing E (Al_18_Mg_3_Cr_2_) dispersoids and quench-induced *η* Mg(Zn,Cu,Al)_2_ at the interface of E (Al_18_Mg_3_Cr_2_) with the matrix during processing.
5. Susceptibility to corrosion attack after 24 h immersion in a pH 1 solution decreases with 0.1 wt.% Cr addition due to decreased fraction of HAGBs to LAGBs and the depletion of solute elements diffusing to the grain boundaries to form precipitates resulting from the formation of Cu- and Zn-containing E (Al_18_Mg_3_Cr_2_) dispersoids and the quench-induced *η* Mg(Zn,Mg,Cu)_2_ phase. Corrosion mechanisms are a combination of IGC and grain attack, and no mechanism change occurs with 0.1 wt.% Cr addition.

## Figures and Tables

**Table 1:** Composition of alloys (in wt.%).

Composition	Al	Zn	Mg	Cu	Zr	Ti	Fe	Si	Cr
7449	Bal.	7.63	1.97	1.72	0.11	0.027	0.08	0.06	-
7449+Cr	Bal.	7.63	1.97	1.64	0.10	0.027	0.08	0.10	0.10

## Data Availability

The raw/processed data required to reproduce these findings cannot be shared at this time as the data also forms part of an ongoing study.
